# Supplementary material for: Localizing Post-Admixture Adaptive Variants with Object Detection on Ancestry-Painted Chromosomes
Source: Mol Biol Evol. 2023 Mar 22;40(4):msad074. doi: 10.1093/molbev/msad074 (PMC10116606; doi:10.1093/molbev/msad074)
Supplement: msad074_Supplementary_Data [file msad074_supplementary_data.pdf]

Supplementary Figures 1-5 & Tables 1-2

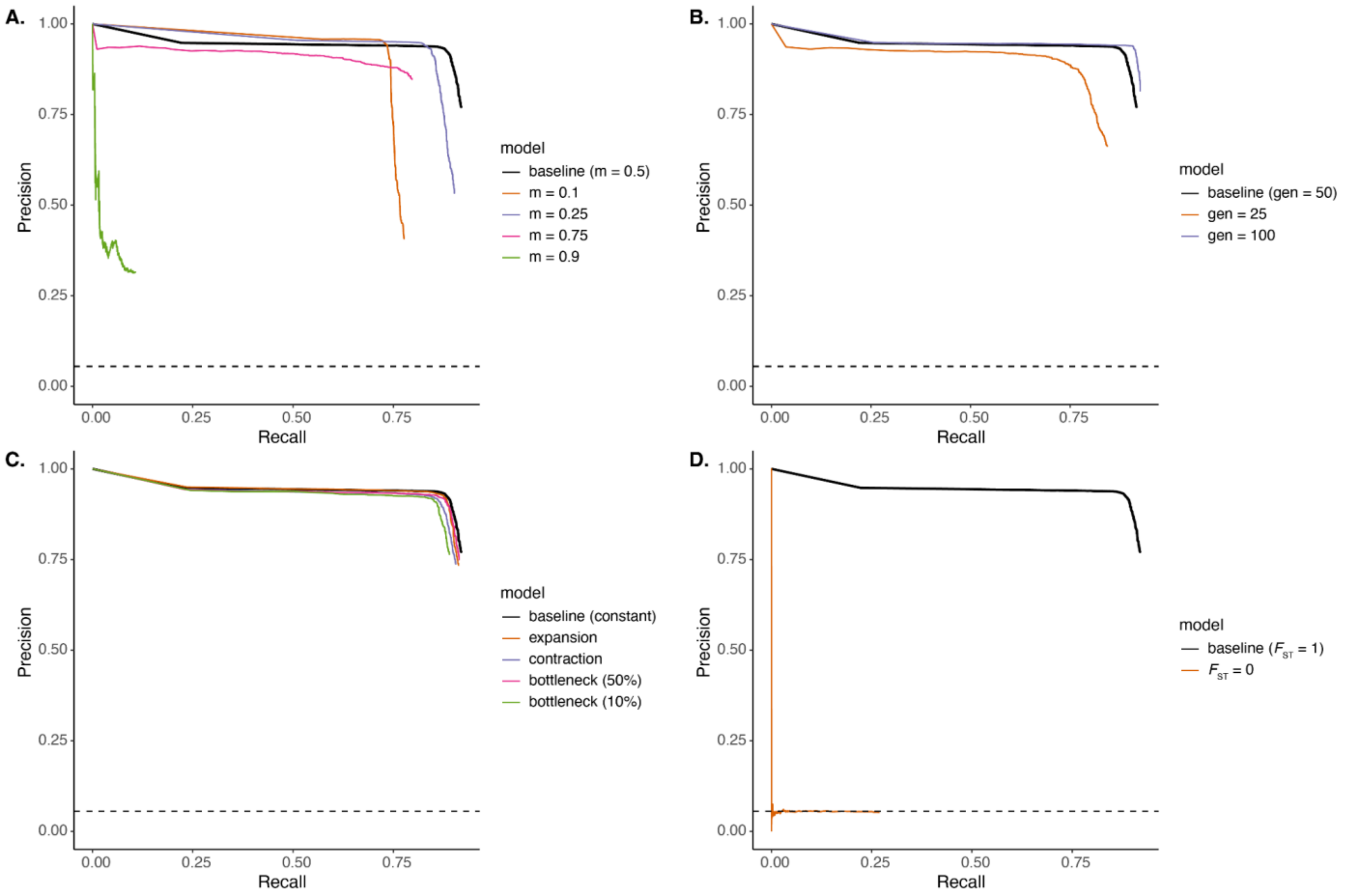

Supplemental Figure 1. Precision-Recall curves comparing performance under demographic model misspecifications to the baseline scenario for high resolution full ancestry images; baseline is the solid black line in each plot. Panels show different categories of misspecification: A) founding admixture contribution from the population providing the beneficial allele, B) number of generations since admixture occurred, C) population size change since the founding of the admixed populations, and D) level of differentiation between the source populations for the variant under selection. Area under the curves (AUC) can be found in Table 2. The no-skill classifier is indicated by the dashed black lines in each plot.

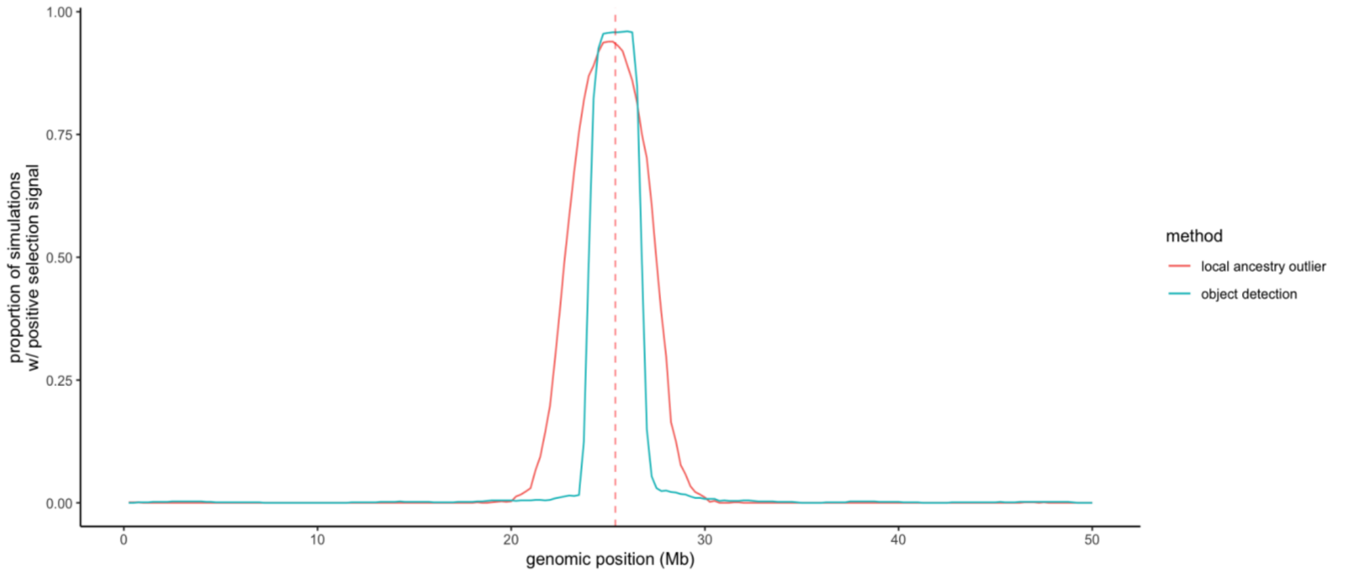

Supplemental Figure 2. Comparison of local ancestry outlier approach and object detection method. Replot of data from Figure 3A, showing, for each genomic window, the proportion of simulations that had that region classified as “under selection” by either the object detection or local ancestry outlier methods.

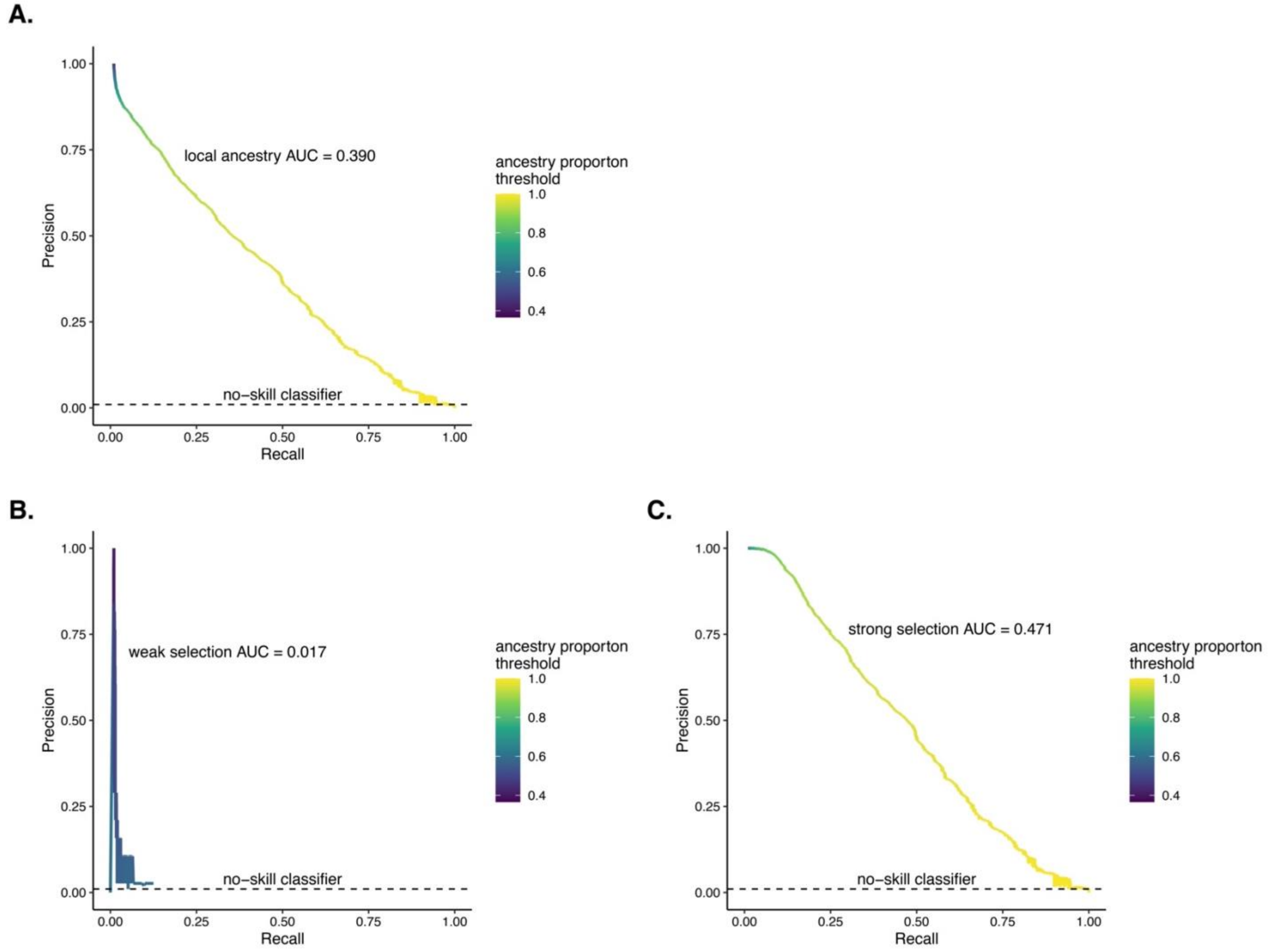

Supplemental Figure 3. Alternative measure of performance of local ancestry outlier approach. We used the same simulations that were generated for Figure 3 over a range of selection coefficients. We defined the “prediction score” as the ancestry proportion, and calculated PR over the range of local ancestry proportions (~0.367 to ~1). Because the “selected variant” is at the very edge of the 100th window, we labeled both windows 100 and 101 as “positives” and everything else as negatives. (A) across selection coefficients. (B) Splitting into “weak selection” simulations (s < 0.01, n = 3800 [200 windows for 19 simulations]) and (C) “strong selection” simulations (s > 0.1), n = 162600 [200 windows for 813 simulations]). Evaluating performance in this way punishes the local ancestry method more than Figure 3 because the wide affected region with high ancestry proportion results in low recall over a range of outlier “thresholds.”

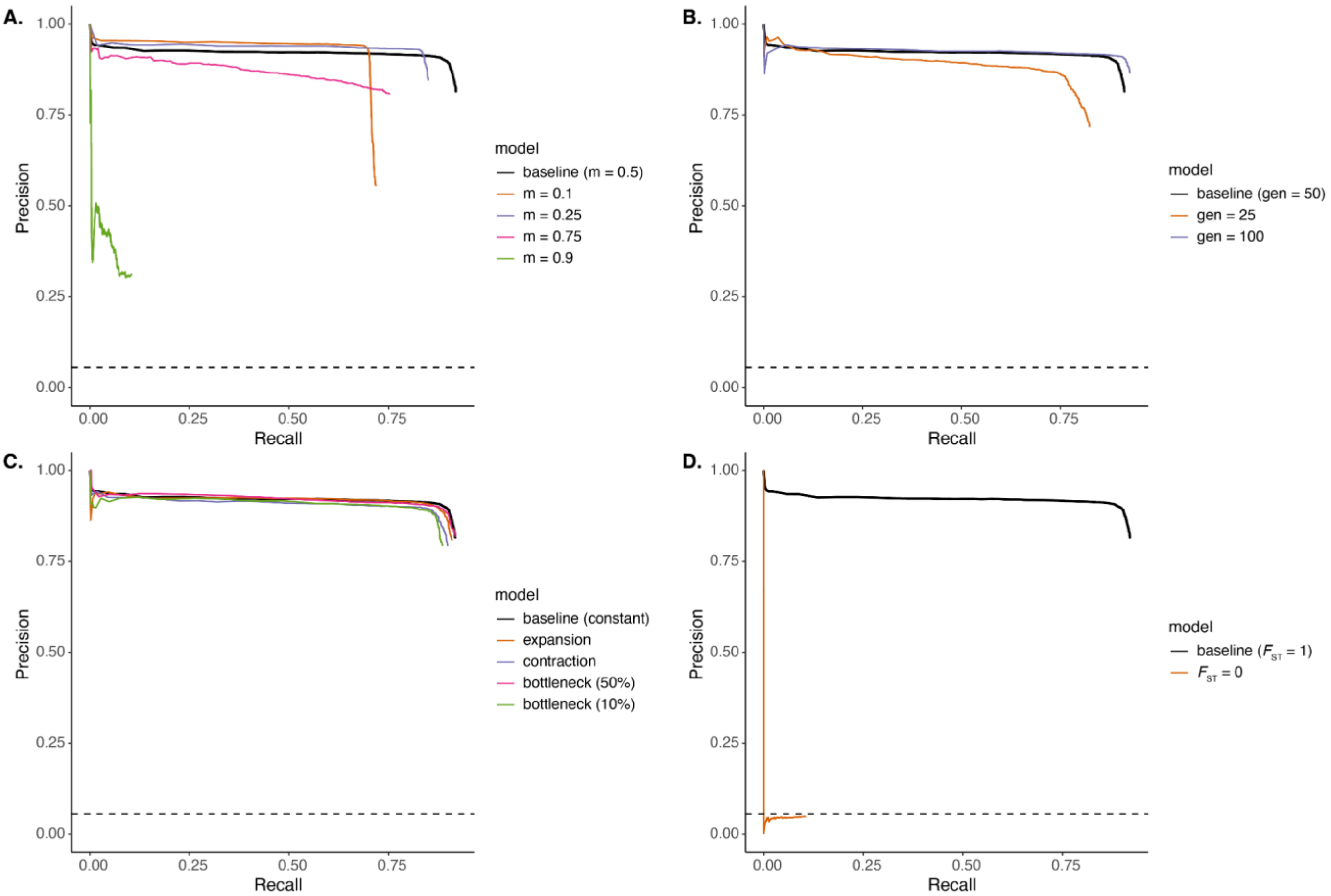

Supplemental Figure 4. Precision-Recall curves comparing performance under demographic model misspecifications to the baseline scenario (i.e. the scenario that the network was trained on) for low-resolution ancestry resolution images; baseline is the solid black line in each plot. Panels show different categories of misspecification: A) founding admixture contribution from the population providing the beneficial allele, B) number of generations since admixture occurred, C) population size change since the founding of the admixed populations, and D) level of differentiation between the source populations for the variant under selection. Area under the curves (AUC) can be found in Table S2. The no-skill classifier is indicated by the dashed black lines in each plot. Analogous to Figure S1 for high-resolution ancestry.

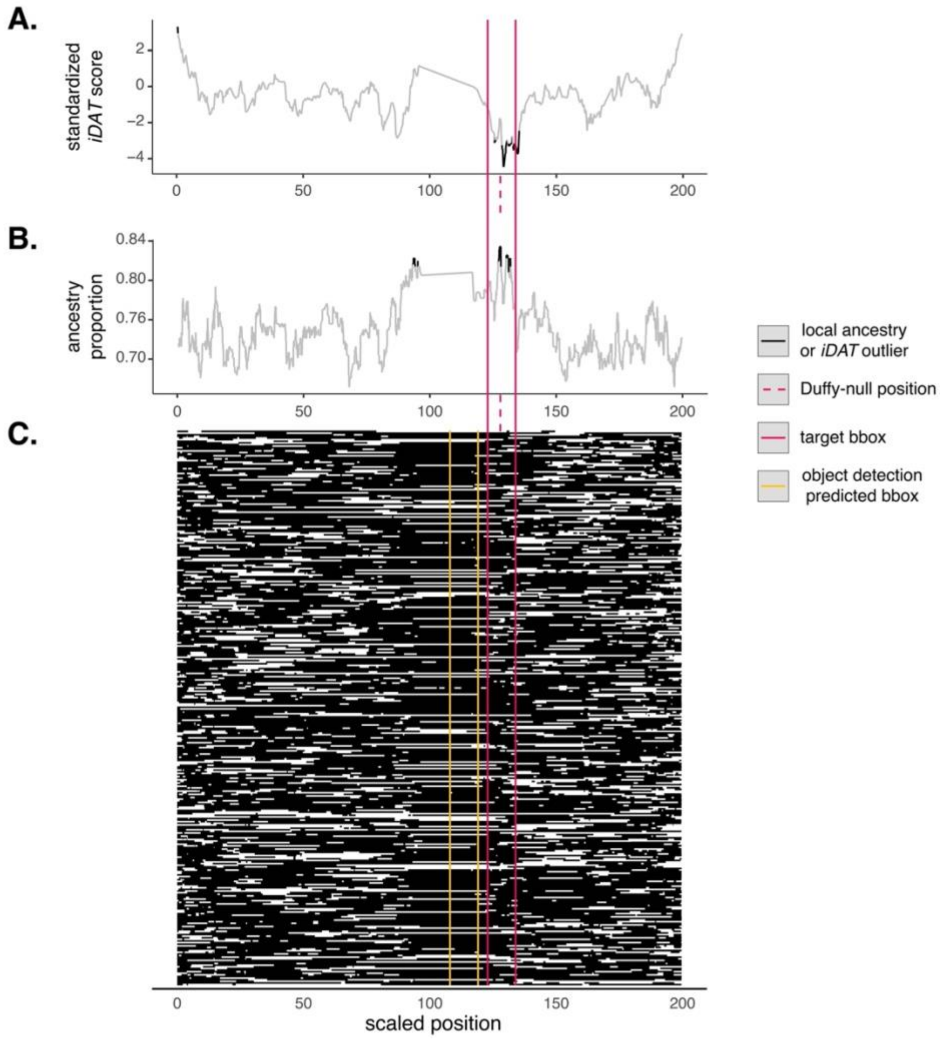

Supplemental Figure 5. Analogous to Figure 4 using physical rather than genetic distances. Identification of a known adaptive allele in a human population using multiple ancestry-based methods. We compare multiple methods to detect a well-known example of post-admixture positive selection in the admixed human populations from Santiago, Cabo Verde on the Duffy-null allele protective against *P. vivax* malaria (Hamid et al., 2021). (A) *iDAT* from Hamid et al., 2021, (B) ancestry outlier detection using a 3 standard deviation cutoff, and (C) the object detection approach developed in this paper. African ancestry in black and European ancestry in white. The image represents the entirety of chromosome 1 for 172 individuals. The dashed line indicates the position of the adaptive allele. The inferred bbox using object detection (C) is in yellow, closely matching the true bbox centered on the adaptive allele (red) in size and location. The other two methods infer multiple and/or longer regions as potentially under selection. Using physical distance, object detection identifies a single adaptive (yellow) variant near but not overlapping the true allele (red).

ancestry resolution	bbox detection rate	average width	average number of bounding boxes	precision	recall
high (full ancestry)	0.861	4.956 (var: 0.264, n=8561)	1.033 (var: 0.160, n = 9000)	0.768	0.756

Supplemental Table 1. Performance of object detection method with a smaller 5-pixel bbox using 800 training images and 200 validation images.

Supplemental Table 2. Performance of object detection method on images generated from demographic misspecifications for low resolution ancestry. Further details of models in Materials and Methods, Figure S4.

misspecification	bbox detection rate	average width	average number of bboxes	precision	recall	AUC
none (baseline)	0.950	10.834 (var = 0.580, n = 1964)	1.0175 (var = 0.064, n = 2000)	0.867	0.870	0.811
m = 0.1	0.723	10.778 (var = 0.793, n = 824)	0.895 (var = 0.283, n = 1000)	0.786	0.675	0.649
m = 0.25	0.857	10.819 (var = 0.649, n = 862)	0.872 (var = 0.135, n = 1000)	0.922	0.798	0.764
m = 0.75	0.788	10.843 (var = 0.638, n = 839)	0.841 (var = 0.138, n = 1000)	0.821	0.690	0.630
m = 0.9	0.073	10.994 (var = 0.080, n = 194)	0.194 (var = 0.156, n = 1000)	0.332	0.065	0.040
gen = 25	0.860	10.823 (var = 0.597, n = 946)	0.987 (var = 0.119, n = 1000)	0.793	0.768	0.711
gen = 100	0.970	10.786 (var = 0.805, n = 993)	1.012 (var = 0.038, n = 1000)	0.886	0.887	0.827
Fst = 0	0.032	10.867 (var = 0.271, n = 635)	1.073 (var = 1.080, n = 1000)	0.047	0.042	0.004
bottleneck (50%)	0.947	10.857 (var = 0.491, n = 988)	1.037 (var = 0.094, n = 1000)	0.853	0.868	0.812
bottleneck (10%)	0.931	10.846 (var = 0.524, n = 989)	1.022 (var = 0.062, n = 1000)	0.835	0.842	0.774
expansion	0.939	10.823 (var = 0.632, n = 984)	1.028 (var = 0.081, n = 1000)	0.856	0.863	0.802
contraction	0.938	10.873 (var = 0.419, n = 984)	1.016 (var = 0.068, n = 1000)	0.847	0.848	0.785
